# COVID-19 related stressors and mental health outcomes of expatriates in international construction

**DOI:** 10.3389/fpubh.2022.961726

**Published:** 2022-07-15

**Authors:** Lili Gao, Xiaopeng Deng, Weimin Yang, Jie Fang

**Affiliations:** ^1^Department of Construction and Real Estate, Southeast University, Nanjing, China; ^2^Department of Architecture and Civil Engineering, City University of Hong Kong, Kowloon, Hong Kong SAR, China; ^3^School of Trade and Logistics, Jiangsu Vocational Institute of Commerce, Nanjing, China; ^4^SINOPEC Engineering (Group) Co. Ltd. (SEG), Beijing, China

**Keywords:** COVID-19 related stressors, psychological resilience, mental health, international assignments experience, expatriates, international construction

## Abstract

The construction industry is labor-intensive, and employees' mental health has a significant impact on occupational health and job performance. In particular, expatriates in international projects under the normalization of the epidemic are under greater pressure than domestic project employees. This paper aims to explore the association of stressors and mental health in international constructions during COVID-19. Furthermore, test the mediation effect of psychological resilience and moderating effort of international experience in this relationship. A survey of 3,091 expatriates in international construction projects was conducted. A moderating mediation model was employed to test the effect of psychological resilience and international experience. Then, statistical analysis with a bootstrap sample was used to test the mediation effect of the model, and a simple slope was used to test the moderating effect. Moderated by experience, the slope of the effect of stressors on psychological resilience changed from −1.851 to −1.323. And the slope of the effect of psychological resilience on mental health outcomes reduced by about 0.1. This suggests that experience is one of the buffering factors for individual psychological resilience of expatriates to regulate stress. Theoretically, this study verifies the mediation effect of psychological resilience between COVID-19 related stressors and mental health outcomes and importance of an expatriate's experience in an international assignment. Practically, this study provides guidelines for international construction enterprises and managers to make an assistant plan for expatriates during this pandemic time and pay more attention to their psychological status. The research also suggests that the best choice for challenging assignments is choosing a more experienced employee.

## Introduction

Since January 2020, COVID-19 has threatened people's physical and mental health worldwide. According to the World Health Organization (WHO), as of December 31, 2021, COVID-19 has caused 285,685,390 infections and 5,430,101 deaths worldwide (1). Previous studies have shown increased levels of psychological distress and perceived mental illness in different populations during pandemics (2–6) and large-scale disasters (7, 8). Studies have shown that people have higher depression, anxiety, and stress levels during the COVID-19 pandemic than usual (3, 9). Some studies have also found that everyone's reaction to COVID-19 is different (10). Some people can quickly adapt to this sudden situation, and some cannot. Previous studies have shown that individuals' characteristics and abilities may be responsible for different outcomes in coping with a crisis (11), such as individual psychological resilience. People with high levels of psychological resilience can better adapt to the influence of COVID-19 (12).

The countries' response to COVID-19 has entered a period of normalization. Employees in various industries are trying their best to resume production and work, and the international construction contracting industry is recovering fully (13). Different from other industries, international construction business takes place overseas, and international construction enterprises need to send many employees to the host country to work. Taking Chinese contractors as an example, a total of 119,000 people was dispatched to overseas project contracting work in 2021, according to the Chinese Ministry of Commerce (14). In addition to adapting to common cultural differences (15, 16), expatriates also need to adapt to the differences in epidemic prevention in the post-epidemic period. The Chinese government's defense policy against COVID-19 is “dynamic clearing,” which is different from “coexisting with the virus” in many countries in the world. The difference makes many Chinese expatriates uncomfortable with the local epidemic prevention and control after working overseas and even anxiety.

International construction is inherently a high-risk industry for international contractors (17–19). For example, a car bomb attack in Pakistan killed 9 Chinese construction workers in 2021. Managers of international projects should always pay attention to the threat of such emergencies to expatriates. At the same time, the cost of sending employees by international construction companies is very high (20, 21), especially in this particular period. If an expatriate cannot adapt well after arriving in the host country, has physical and mental problems, or even wants to return to the country in advance, the company needs to incur extra costs such as high airfare and isolation fees. Poor assignments or failures negatively impact project performance (22, 23). Therefore, the enterprise managers and international construction project managers urgently hope that the expatriates can adapt to the expatriate work safely, healthily, and quickly and complete the established tasks efficiently. Understanding expatriates' physical and mental state in the host country and understanding what factors affect their mental health performance can be very meaningful for international construction managers to help them develop a help plan. However, the existing researches have not paid much attention on the group of expatriates. Especially, there is a lack of understanding in the context of international construction.

**Figure 1:**
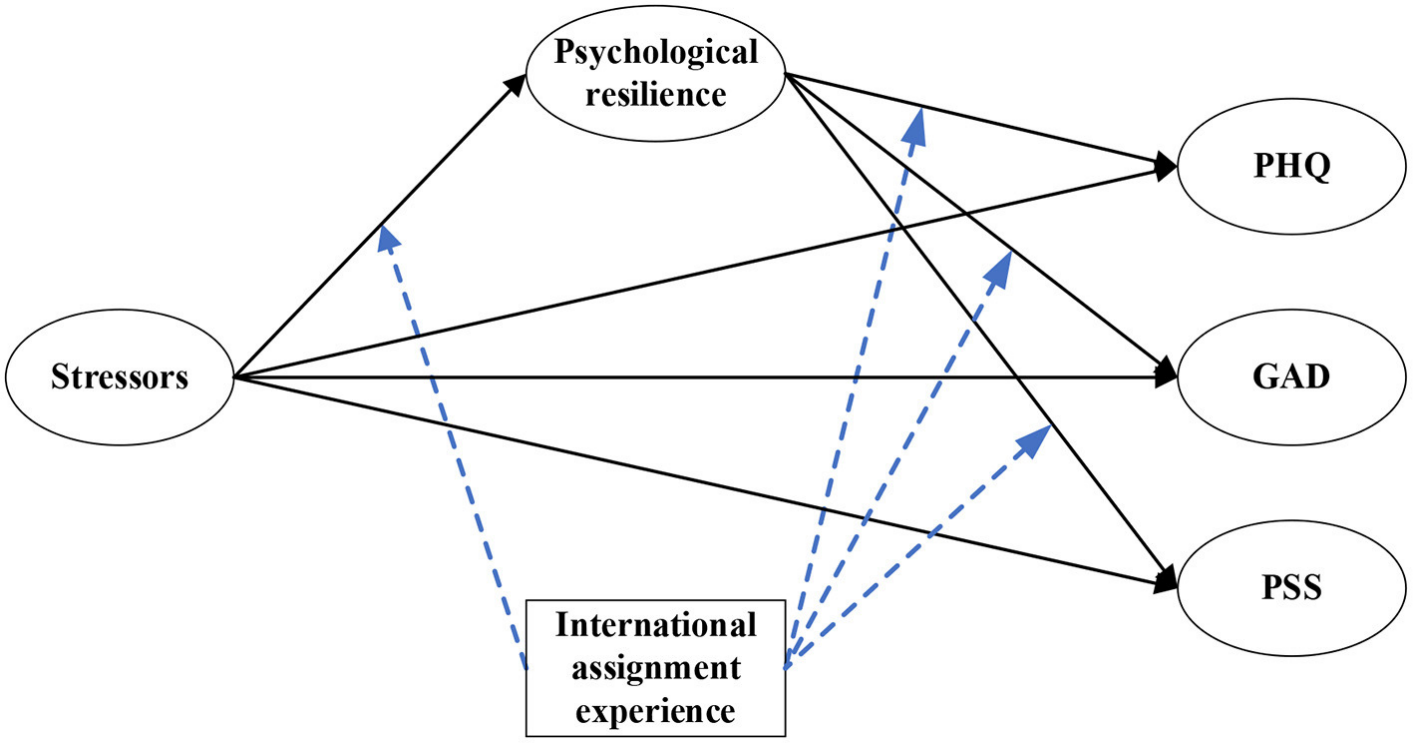
Hypothesis model.

This study aimed to analyze the impact of COVID-19 related stressors on international construction expatriates' mental health levels (depression, anxiety, perceived stress). By constructing a model (Figure 1) mediated by individual psychological resilience and moderated by expatriate experience, this study uses questionnaires to investigate and analyze expatriates of international contractors. Details of the development of the hypotheses are presented in the following section.

## Theory and hypotheses

### Psychological resilience, stressors, and mental health

There have been many pieces of research on psychological resilience. Most of them focus on the antecedents and consequences of psychological resilience or take psychological resilience as an intermediary or regulatory variable. The psychological resilience of this paper refers to the individual in the workplace situation. There is no uniform definition of psychological resilience. However, they all believe that the experience of adversity is the first defining element (24). This paper defines psychological resilience from the perspective of ability (25), explicitly referring to the ability of expatriates to deal with stressors and adjust themselves to normal status. Fisher, Ragsdale (26) point stressors at work may be short-term and sudden high-risk events (e.g., public safety events) or long-term continuous circumstances (e.g., work stress). For expatriates from international construction companies, they have to face the public workplace stressors mentioned above. At the same time, due to the high risk of the international construction industry and the working environment of uprooting, especially the impact of COVID-19, stressors include both from the pandemic (27), family (28), and workplace (29).

In workplace, stressors are regarded as an adversity, and individual's protective resources are first invoked in response to stress (30). Protective resources are directly related to psychological resilience (31). If there are too many stress events and the resources that individuals can use are insufficient, the psychological elasticity will be worse. Taking mental health as an outcome of psychological resilience, scholars found that employees' resilience is positively related to their mental health (32), and negatively affects the expression of burnout and emotional exhaustion (33–35). McLarnon and Rothstein (36) pointed out that half of the indicators in the Workplace Resilience Scale were negatively correlated with depression. Moreover, Ferris, Sinclair (37) emphasized that the lack of individual psychological resilience caused physical and psychological stress, such as low emotional, easy to fatigue, and poor attention. Previous studies have started investigating the individual resilience as a mediation factor. The challenge-hindrance stressors model posits that workplace stressors can be grouped into two categories. Hindrance stressors will interfere with performance or goals, while challenge stressors contribute to performance opportunities (38). Based on the model, Crane and Searle (39) found resilience played a full mediation effect between the negative relationship of challenge stressors and strain and the positive relationship of hindrance stressors and strain. Also, Kinman and Grant (32) found resilience played a full mediation effect between the negative relationship of emotional intelligence and mental distress.

Since the outbreak of COVID-19, some studies focus on the relationship between resilience and mental health. However, researchers have regarded resilience as one of the components of psychological capital, which has been found to be related with factors in workplace and employee's mental health (25). Lawal et al. (40) used the standard scale to test the mental health of ordinary people in COVID-19. Their research pointed out that the individuals' psychological distress, depression, and anxiety were significantly higher than those of normal ones due to the influence of COVID-19. In the early study, researchers paid more attention to doctors, nurses, and other people who had direct contact with COVID-19 (41). Rossi et al. (42) analyzed the mental health status of Italian residents during the closed period. They found that stressors in COVID-19 significantly impacted depression, anxiety, and stress perception. Barzilay, Moore (43) found that people with higher psychological resilience were not prone to depression and anxiety. As the pandemic continues, we believe this phenomenon may also be present among international construction expatriates. Therefore, the hypothesis is proposed.

*Hypothesis 1: Psychological resilience mediates the influence of stressors during COVID-19 on expatriates' mental health*.
*Hypothesis 1a: Psychological resilience mediates the influence of stressors during COVID-19 on expatriates' level of depression*.
*Hypothesis 1b: Psychological resilience mediates the influence of stressors during COVID-19 on expatriates' level of anxiety*.
*Hypothesis 1c: Psychological resilience mediates the influence of stressors during COVID-19 on expatriates' perceived stress levels*.

### International assignments experience and psychological resilience

Many studies have confirmed that psychological resilience among individuals differs, which depends on many factors. Personal resources are considered one of the most critical factors (44). Employees' professional knowledge about the work (45) or technology related to work (46) is positively related to psychological resilience. Although there is no direct research on the relationship between work experience and psychological resilience, like workability, work experience is also an essential resource in the individual workplace. Experienced workers know better how to deal with difficulties and perform better under stressors (47). The positive state and emotion could improve employees' psychological resilience, while the negative emotions had an opposite effect in case of an organizational crisis (48). Therefore, we believe that it is reasonable that expatriate experience, as a unique resource, is related to individual psychological resilience. Therefore, the hypothesis is proposed.

*Hypothesis 2: International assignment experience moderates the relationship between stressors during COVID-19 and psychological resilience. When expatriates' international assignment experience is more prosperous, the negative effect of stressors during COVID-19 on expatriates' psychological resilience is weaker*.

### International assignments experience and mental health

Resilience is a protective mechanism when people are facing of adversity which is usually associated with lower mental distress (49). However, no thorough research points out that work experience is directly related to individual mental health statuses such as depression and anxiety. However, Wiseman, Curtis (50) found that individuals with different life experiences have different manifestations of depression and anxiety. Stress levels were higher for the general public than those working directly (front line nurses) with COVID patients, possibly related to experience and confidence (51).

Moreover, Rossi et al. (12) and Nwachukwu et al. (52) found that in the elderly group, psychological resilience has a more significant impact on individual mental health states such as depression and anxiety. It is found that age is the regulatory factor between psychological resilience and mental health. Furthermore, generally, older people have more work experience. Therefore, the hypothesizes are proposed.

*Hypothesis 3: International assignment experience moderates the indirect effect of stressors during COVID-19 on expatriates' mental health via psychological resilience. When expatriates' international assignment experience is less prosperous, the negative effect of expatriates' psychological resilience on mental health is more robust*.
*Hypothesis 3a: International assignment experience moderates the path of psychological resilience on expatriates' level of depression*.
*Hypothesis 3b: International assignment experience moderates the path of psychological resilience on expatriates' level of anxiety*.
*Hypothesis 3c: International assignment experience moderates the path of psychological resilience on expatriates' perceived stress level*.

## Materials and methods

### Participants and procedure

We tested the hypothesis model with a sample of expatriates in international construction enterprises. In the study, expatriates were defined as “Citizens of the home country or third country whom international construction companies appoint to work in the host country, among which the citizens of the home country who work in the host country are mainly.” Therefore, we cooperated with SINOPEC Engineering (Group) Co. Ltd. (SEG), which was listed in the Top 250 contractors of ENR in 2020. We used the online questionnaire platform of Wenjuanxing to collect data. The HR department sent the questionnaire to all the expatriates by the inner system of SEG from May 5 to May 25 in 2020. In the survey, 3,091 valid questionnaires were received, including managers, workers, subcontractors, and others. The characteristics, including gender, age, position, and international assignments experience, were collected in the demographic information (Table 1). The expatriates of enterprises participating in the research are informed in advance that the results are only used for academic research and participate voluntarily.

**Table 1:** Demographic characteristics of expatriates (*N* = 3,091).

**Characteristics**	**Number**	**Percentage**
Gender
Male	3,021	97.7
Female	70	2.3
Age
21–30 years	233	7.2
31–40 years	1,515	49
41–50 years	1,136	36.8
≥51 years	217	7
Position
Managers	690	22.3
Workers	1,866	60.4
Subcontractors	333	10.8
Others	202	6.5
**International assignments experience**
≤ 1 year	218	7.1
1–3 years	425	13.7
3–5 years	590	19.1
5–10 years	915	29.6
≥10 years	943	30.5

### Measures

Stressors (SE) during COVID-19 were assessed using a checklist of stress events developed by this research. The list of stress events was obtained through a literature review and employee interviews. Therefore, the checklist explores thirteen different stressors in Table 2. In the questionnaire, each item has a yes/no response as a binary variable. 0 = “feel no stress due to this during COVID-19” and 1 = “feel stress due to this during COVID-19.” The Cronbach's alpha was 0.888 in this research.

**Table 2:** The checklist of stress events related to COVID-19.

**Code**	**Construct and items**	**Sources**
SE01	Being unable to return to China	Driessen (53)
SE02	High work pressure	
SE03	Uncertainty about the development of the epidemic	
SE04	Anxiety, and worry of family members and themselves	
SE05	Discrimination and prejudice in the host country	
SE06	Worry about being infected	Tripathi and Singh (27)
SE07	Worry about similar symptoms such as a cold and fever	
SE08	Worry about people around them being infected	
SE09	Worry about the epidemic prevention and control measures in the host country are not effective enough	
SE10	Suffer from chronic diseases	Al Maskari, Al Blushi (54)
SE11	Conflict, and trouble in family relations	Shah, de Oliveira (28)
SE12	Overseas workplace safety management pressure	Shaaban (29)
SE13	Overseas public safety pressure	

Psychological resilience (PR) was measured by 10-item Connor–Davidson Resilience Scale (CD-RISC-10). The CD-RISC is used widely to assess resilience (55). The original version of the CD-RISC-10 was created by Campbell-Sills and Stein (56) includes ten items. The response scale has a 5-point range: 1 (not true at all), 2 (rarely true), 3 (sometimes true), 4 (often true), and 5 (true nearly all of the time). In the present study, Cronbach's alpha for the overall CD-RISC-10 was 0.937.

Depression was measured by the 9-item Patient Health Questionnaire (PHQ-9) in a Chinese version (57). PHQ-9 contains nine items measured by a 4-point Likert scale. The total score of PHQ-9 was analyzed as a continuous variable. PHQ-9 is used as a tool for screening depression in many countries around the world. The Cronbach's alpha was 0.938 in this research.

Anxiety was measured by the 7-item Generalized Anxiety Disorder questionnaire (GAD-7) in a Chinese version (58). GAD-7 contains seven items measured by a 4-point Likert scale. The total score of GAD-7 was analyzed as a continuous variable. Many countries use GAD-7 for anxiety screening. In this research, Cronbach's alpha was 0.936.

Perceived stress was assessed by the Chinese version of the 10-item Perceived Stress Scale (PSS-10) (59). PSS includes ten items rated on a 5-point Likert scale. The Cronbach's alpha was 0.922 in this research.

International assignments experience was divided into five groups, and respondents could choose the answer according to their situation. This variable was analyzed as a ordinal variable with five scores (1 = 0–1 years, 2 = 1–3 years, 3 = 3–5 years, 4 = 5–10 years, 5 = 10 years and over.) The scales and question items are listed in Appendix A.

### Data analysis

After standardized the data of PHQ and GAD scales into 5-point, the analysis process followed the steps below. Firstly, we used Harman's single-factor test to check the common method biases of all of the items in the four scales. Moreover, confirmatory factor analyses (CFA) were conducted using AMOS 26.0. Secondly, we used the SPSS 26.0 to conduct descriptive statistics and correlation analysis. Thirdly, verify the mediating effect between stressors and mental distress with psychological resilience as the mediating variable. The significance of mediation effect was judged by checking whether the confidence intervals of 95% bootstrap repeated 5,000 times included zero. Finally, conditional indirect effects of COVID-19-related stressors on mental distress, which was mediated by psychological resilience and moderated by international assignment experience was tested. This moderated mediation model is based on Hayes's Model 58. Moreover, we conducted the simple slope test to determine how the international assignment experience moderates the relationship between COVID-19 related stressors and psychological resilience.

## Results

### Assessment of common method bias and confirmatory factor analysis

We randomly divided the sample into two parts. Half of the samples were used for exploratory factor analysis (EFA) without rotation. The results of Harman's single-factor test extracted ten factors with eigenvalues above one. Before the rotation the variance explained by the leading common factor was 37.34%, which was less than the 40% required by the critical criteria (60). Therefore, this study does not consider the influence of common method bias.

The other half of samples was conducted a confirmatory factor analysis in Amos 26.0. The expatriates' international assignments experience and stressors questionnaire answers objective facts and does not test an implicit variable. Therefore, the scale's content validity and discriminant validity are not tested. As shown in Table 3, all factor loadings were above 0.6 and significant, indicating that the measured item validity was acceptable. The composite reliability (CR) for each construct's was >0.7, indicating that CR was acceptable. Moreover, each construct's average variance extracted (AVE) is more significant than 0.5, indicating that convergence validity was acceptable. In addition, the square root of AVE of each construct is larger than the correlation coefficient between any two constructs. This shows that the discrimination of each structure is significant. Therefore, the validity of each measure is acceptable. And the RMSEA value of the model is 0.062 (< 0.01), CFI value is 0.937 (>0.09), and NFI value is 0.933 (>0.09). Thus, the model fit is acceptable.

**Table 3:** Results of confirmatory factor analysis of each measure.

**Variable**	**Estimate**	**CR**	**AVE**	**1**	**2**	**3**	**4**
1.PR	0.699–0.851	0.930	0.579	0.761			
2.PHQ	0.651–0.879	0.946	0.667	−0.665	0.817		
3.GAD	0.866–0.905	0.956	0.755	−0.619	0.773	0.869	
4.PSS	0.718–0.897	0.923	0.668	−0.510	0.658	0.570	0.817

*CR, composite reliability; AVE, average variance extracted. The discriminatory validity value of each construct is shown along the diagonal in bold*.

### Descriptive statistics and correlational

Means, standard deviations, and correlations of the main study variables were presented in Table 4. Correlation analyses showed that expatriates' psychological resilience is significantly associated with stressors (*r* = −0.374, *p* < 0.01), PHQ (*r* = −0.665, *p* < 0.01), GAD (*r* = −0.619, *p* < 0.01), and PSS (*r* = −0.510, *p* < 0.01). This satisfied the prerequisites of mediation analysis.

**Table 4:** Descriptive statistics and correlations between variables.

**Variable**	**Mean**	**SD**	**Age**	**Gender**	**IAE**	**SE**	**PR**	**PHQ**	**GAD**	**PSS**
Age	2.436	0.729	1.000							
Gender	1.023	0.149	−0.088^**^	1.000						
IAE	3.628	1.242	0.423^**^	−0.086^**^	1.000					
SE	0.374	0.172	−0.019	−0.030	−0.029	1.000				
PR	4.214	0.735	0.120^**^	0.044^*^	0.114^**^	−0.374^**^	1.000			
PHQ	1.684	0.680	−0.155^**^	−0.013	−0.099^**^	0.365^**^	−0.665^**^	1.000		
GAD	1.717	0.829	−0.118^**^	−0.024	−0.104^**^	0.364^**^	−0.619^**^	0.773^**^	1.000	
PSS	2.290	0.767	−0.055^**^	0.005	−0.026	0.320^**^	−0.510^**^	0.658^**^	0.570^**^	1.000

*N = 3,091; Age: 1 = 21–30 years, 2 = 31–40 years, 3 = 41–50 years, 4 = 51 years and over; Gender: 1 = Male, 2 = Female; work experience: 1 = 0–5 years, 2 = 6–10 years, 3 = 11 years and over; IAE (International Assignments Experience): 1 = 0–1 years, 2 = 1–3 years, 3 = 3–5 years, 4 = 5–10 years, 5 = 10 years and over*.

** *p < 0.01*,

* *p < 0.05*.

For the control variables, age correlated significantly with IAE (*r* = 0.423, *p* < 0.01), PR (*r* = 0.120, *p* < 0.01), PHQ (*r* = −0.155, *p* < 0.01), GAD (*r* = −0.118, *p* < 0.01), and PSS (*r* = −0.055, *p* < 0.01); gender correlated significantly with IAE (*r* = −0.086, *p* < 0.01) and PR (*r* = 0.044, *p* < 0.05). In the tests of relevant hypotheses, age and gender were controlled (61).

### Mediating model analyses

**Figure 2:**
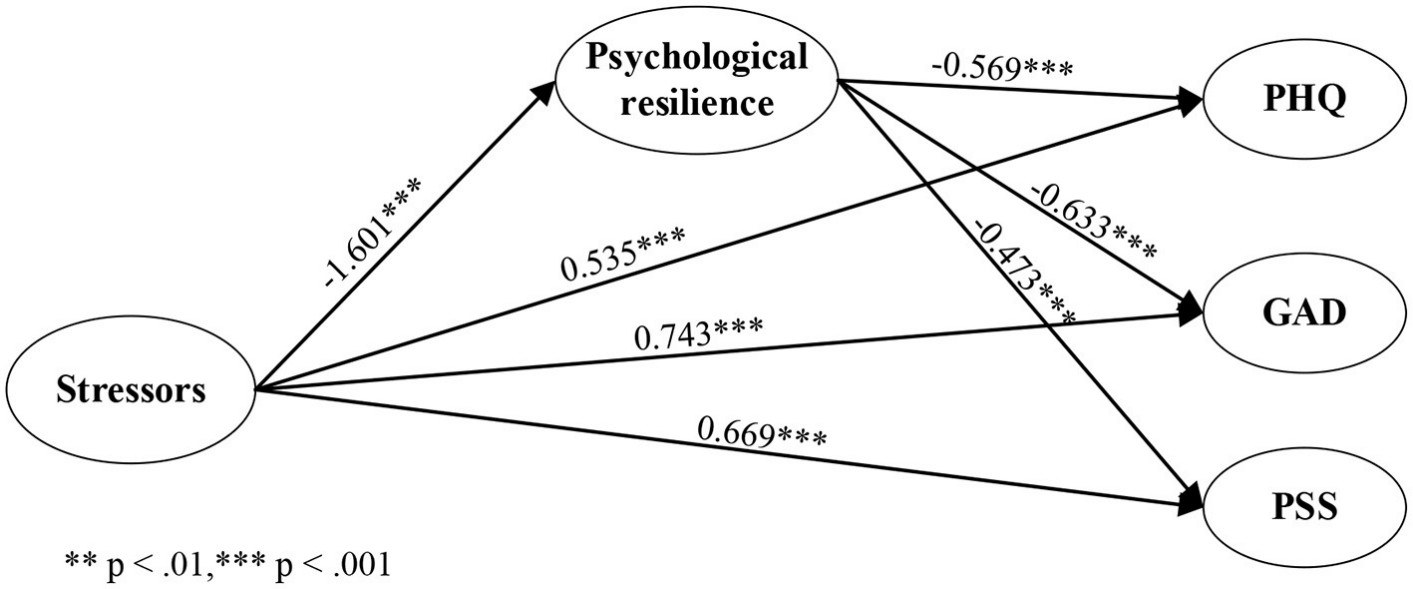
Path coefficients of COVID-19 related stressors, psychological resilience, and mental health.

According to the correlation analysis results, they meet the conditions for establishing an intermediary relationship between the three factors. The mediating model (Hypothesis 1) was tested by Model 4 of PROCESS. The results in Figure 2 show that stressors significantly negatively predicted psychological resilience (*t* = −1.601, *p* < 0.001). When stressors and psychological resilience were considered in the regression equation, psychological resilience was significantly negative with PHQ (*t* = −0.569, *p* < 0.001). The indirect effect was tested by 5,000 resampling bootstraps. Results in Table 5 showed the mediation effect of psychological resilience between COVID-19 stressors and PHQ is significant (Effect = 0.911, SE = 0.056, 95% boot CI = [0.803, 1.022]). The mediation effect's 95% bootstrap confidence interval (CI) did not contain zero, and the indirect effect accounted for 63.26% of the total effect. Therefore, psychological resilience mediated the relationship between stressors and PHQ. Thus, Hypothesis 1a was supported.

**Table 5:** Psychological resilience as a mediator in the relationship between COVID-19 related stressors and mental health.

**Variable**	**Effect**	**Boot SE**	**Boot LL 95% CI**	**Boot UL 95% CI**	**Hypothesis**
PHQ	Total effect	1.445	0.066	1.315	1.577	H1a (Support)
	Direct effect	0.535	0.057	0.424	0.646	
	Indirect effect	0.911	0.056	0.803	1.022	
GAD	Total effect	1.757	0.081	1.598	1.916	H1b (Support)
	Direct effect	0.743	0.072	0.601	0.885	
	Indirect effect	1.013	0.059	0.898	1.128	
PSS	Total effect	1.427	0.076	1.278	1.577	H1c (Support)
	Direct effect	0.669	0.074	0.525	0.814	
	Indirect effect	0.757	0.052	0.660	0.864	

*N = 3,091*.

Also, psychological resilience significantly negatively predicted GAD (*t* = −0.633, *p* < 0.001). Results in Table 5 showed the mediation effect of psychological resilience between COVID-19 stressors and GAD is significant (Effect = 1.013, SE = 0.059, 95% boot CI = [0.898, 1.128]). The mediation effect's 95% bootstrap confidence interval (CI) did not contain zero, and the indirect effect accounted for 57.66% of the total effect. Therefore, psychological resilience mediated the relationship between stressors and GAD. Thus, Hypothesis 1b was supported.

Finally, psychological resilience significantly negatively predicted PSS (*t* = −0.473, *p* < 0.001). Results in Table 5 showed the mediation effect of psychological resilience between COVID-19 stressors and PSS is significant (Effect = 0.757, SE = 0.052, 95% boot CI = [0.660, 0.864]). The mediation effect's 95% bootstrap confidence interval (CI) did not contain zero, and the indirect effect accounted for 53.05% of the total effect. Therefore, psychological resilience mediated the relationship between stressors and PSS. Thus, Hypothesis 1c was supported. The effect value of each path is shown in Figure 2.

### Moderated mediating model analyses

Moderating effects are hypothesized at two stages of the mediation relationship. The first stage is the effect of stressors on psychological resilience. International assignment experience moderates the relationship between stressors during COVID-19 and psychological resilience. When expatriates' international assignment experience is more prosperous, the negative effect of stressors during COVID-19 on expatriates' psychological resilience is weaker. The second stage is the effect of psychological resilience on mental health outcomes. International assignment experience moderates the indirect effect of stressors during COVID-19 on expatriates' mental health via psychological resilience. When expatriates' international assignment experience is less prosperous, the negative effect of expatriates' psychological resilience on mental health is more robust. PROCESS model 58 was conducted to test Hypotheses 2, 3a, 3b, and 3c, respectively.

**Figure 3:**
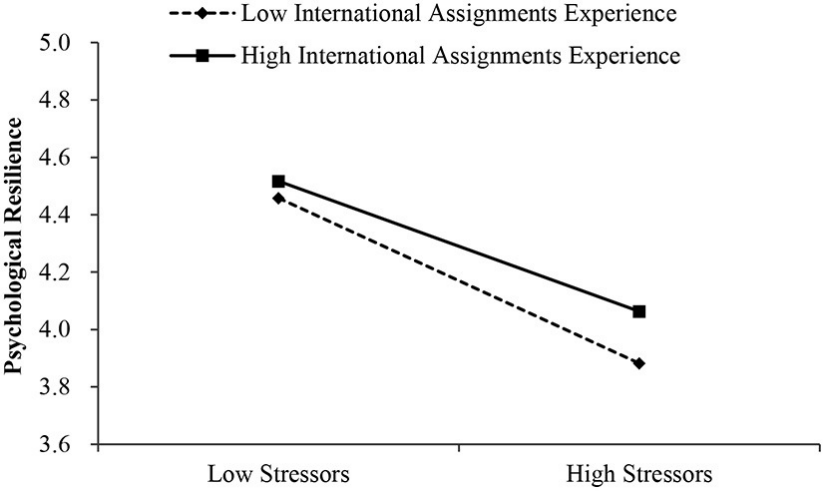
Simple slopes of international assignments experience moderate the relationship between stressors during COVID-19 and psychological resilience.

According to Table 6, it was revealed that effect of stress events during COVID-19 on psychological resilience was significant (Effect = −1.587, SE = 0.071, 95% bootstrap CI = [−1.726, −1.448]), and more importantly, the effect was significantly moderated by expatriates' international assignments experience (Effect = 0.213, SE = 0.057, 95% bootstrap CI = [0.101, 0.324]). For clarity, we plotted stressors during COVID-19 on psychological resilience (Figure 3), separately for low and high stress (Mean-SD and Mean + SD, respectively). Simple slope tests indicate that for expatriates who have high international assignments experience, psychological resilience level is significantly descended as the increase of stressors during COVID-19 (Effect = −1.323, SE = 0.100, *p* < 0.001); for expatriates have low international assignments experience, psychological resilience level is also significantly descended as the increase of stressors during COVID-19 (Effect = −1.851, SE = 0.100, *p* < 0.001). Moderated by experience, the slope of the effect of stressors on psychological resilience changed from −1.851 to −1.323, reduced by about 0.5. It can be revealed that experience is a buffering factor when dealing with stressors during COVID-19, the psychological resilience of expatriates decreases slowly compared with those who have less experience (Figure 3). Thus, H2 was supported.

**Table 6:** International assignment experience modifies the relationship between stressors during COVID-19 and mental health.

**Variable**	**Effect**	**Boot SE**	***t***	***p***	**Boot LL 95%CI**	**Boot UL 95% CI**	**Hypothesis**
Y: PR.							H2 (Support)
X: SE	−1.587	0.071	−22.385	0.000	−1.726	−1.448	
M: IAE	0.060	0.010	6.165	0.000	0.041	0.080	
Interaction: X × M	0.213	0.057	3.728	0.000	0.101	0.324	

*N = 3,091, SE, Stressors; PR, Psychological Resilience; IAE, International Assignment Experience*.

**Figure 4:**
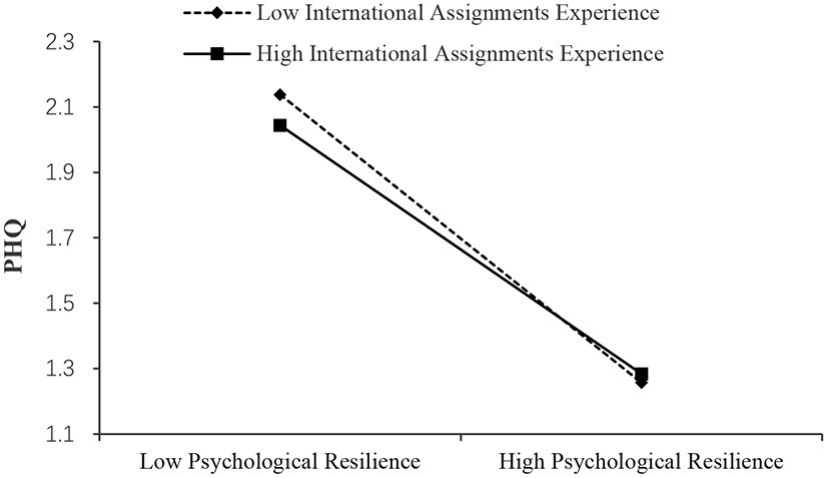
Simple slopes of international assignments experience moderate the effect of psychological resilience on PHQ.

**Figure 5:**
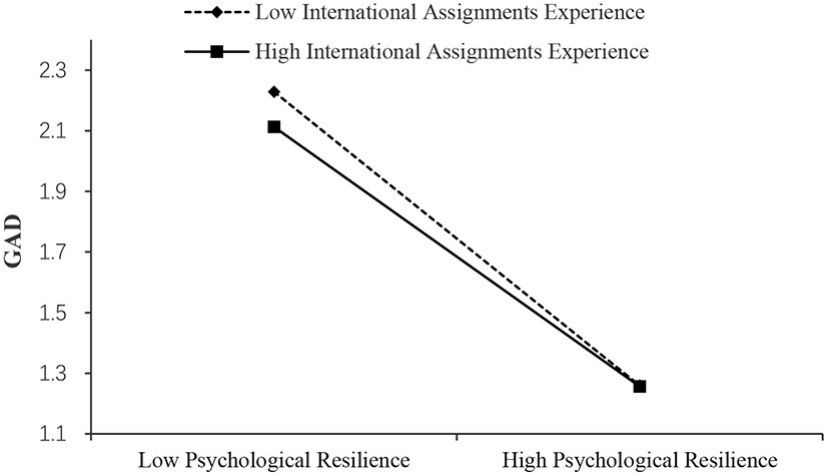
Simple slopes of international assignments experience moderate the effect of psychological resilience on GAD.

**Figure 6:**
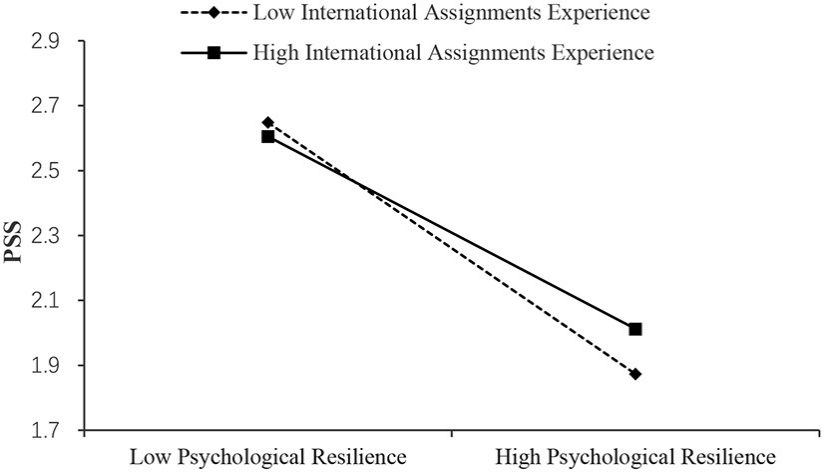
Simple slopes of international assignments experience moderate the effect of psychological resilience on PSS.

As shown in Table 7, the effect of psychological resilience on PHQ was significant when international assignments experience was high (Effect = −0.517, SE = 0.019, 95% boot CI = [−0.555, −0.479]) and low (Effect = −0.599, SE = 0.016, 95% boot CI = [−0.631, −0.567]). And, the of psychological resilience on GAD was significant when international assignments experience was high (Effect = −0.583, SE = 0.025, 95% boot CI = [−0.631, −0.535]) and low (Effect = −0.659, SE = 0.021, 95% boot CI = [−0.700, −0.618]). Also, the effect of psychological resilience on PSS was significant when international assignments experience was high (Effect = −0.403, SE = 0.025, 95% boot CI = [−0.452, −0.354]) and low (Effect = −0.527, SE = 0.021, 95% boot CI = [−0.569, −0.486]). Thus, it can be concluded from the simple slopes that expatriates' international assignments experience attenuated the effect of psychological resilience on mental health (Figures 4–6). Moderated by experience, the slope of the effect of psychological resilience on mental health outcomes reduced by about 0.1. This indicated that experience can be regarded as a buffering factor for individual psychological resilience of expatriates to regulate stress. Thus, H3a, H3b, and H3c were supported. While, the results indicate the buffering effect of experience differs in the two stages. It is more significant between stressors and psychological resilience.

**Table 7:** Results of the moderated path analysis.

**Dependent variables**	**International assignment experience**	**Psychological resilience**→**dependent variables**						**Hypothesis**
		**Effect**	**Boot SE**	***t***	***p***	**Boot LL 95%CI**	**Boot UL 95% CI**	
PHQ	M-SD	−0.599	0.016	−36.672	0.000	−0.631	−0.567	H3a (Support)
	M+SD	−0.517	0.019	−26.780	0.000	−0.555	−0.479	
GAD	M-SD	−0.659	0.021	−31.555	0.000	−0.700	−0.618	H3b (Support)
	M+SD	−0.583	0.025	−23.617	0.000	−0.631	−0.535	
PSS	M-SD	−0.527	0.021	−24.871	0.000	−0.569	−0.486	H3c (Support)
	M+SD	−0.403	0.025	−16.100	0.000	−0.452	−0.354	

*N = 3,091*.

## Discussion

A moderated mediation model was established to assess the indirect relationship between stressors during COVID-19 and mental distress via psychological resilience and whether expatriates' international assignments experience moderated the first and second stages of this indirect association. The results explain how and when stressors during COVID-19 impact expatriates' mental health.

### Mediating effect of psychological resilience

Consistent with our expectation (Hypothesis 1), stressors during COVID-19 positively predicted PHQ, GAD, and PSS scores. Psychological resilience was a mediation factor in this relation, extending previous theory and empirical research. Previous researches showed that the COVID-19 pandemic had significantly increased the depression, anxiety, and stress perceived level (12, 42, 62). Our research findings also confirmed the positive association between stressors and mental distress. Moreover, other than directly affecting depression, anxiety, and stress perceived level, stressors during COVID-19 indirectly affect these three variables. This mediation model suggests a possible reason why the more stressors, the more prone to depression, anxiety, and stress may be beyond their resilience to deal with adversity.

Specifically, it advances our understanding of psychological resilience by applying the negative outcome of COVID-19-related stressors into depression, anxiety, and perceived stress. Although the antecedents and consequences of psychological resilience have been verified with various studies (45, 48, 63), there is no relevant evidence in the expatriates of multinational companies. Consistent with the results of Rossi's et al. (12) research, psychological resilience plays a mediation role between stressors during COVID-19 and mental health. After expatriates encounter COVID-19 related stressors, individual protective factors will play a role in helping expatriates recover from adversity. However, when the resilience is poor, the psychological state of expatriates will change and threaten their mental health. The mental health problems of expatriates in multinational enterprises are more prominent than their domestic employees (64), especially in the face of the outbreak and persistence of COVID-19. In our survey, 70% of the respondents were delayed in their return.

In addition, the two stages of the mediation process will be discussed separately. Consistent with previous reports, we find that COVID-19-related stressors decrease psychological resilience scores (65). According to the standard definition of psychological resilience, individuals experiencing stressors can inspire one's ability to cope with adversities (66). According to resource conservation theory (67, 68), psychological resilience is a positive conservation resource for individuals (69). The more the external stressors, the more resources the individual needs to recover from this state with a longer recovery process. The ability to recover is also reduced (68). Besides, psychological resilience scores are correlated with depression, anxiety, and stress perceived level. It indicates that higher psychological resilience can buffer the effect of COVID-19 related stressors on such depression, anxiety, and stress perceived (70). These researches are consistent with our results that people with a high level of psychological resilience have a more vital ability to recover from adversity. The probability of psychological problems will decrease, and their mental state will be healthier.

Compared with Rossi et al.'s (12) research, in the expatriates' group, the path influence coefficient of stressors during COVID-19 on individual psychological resilience is significantly higher than that of the general group. The path coefficient of COVID-19-related stressors on depression, anxiety, and stress perception is also significantly higher than ordinary people.

### Moderating effect of international assignment experience

The moderating effect on the two stages in our mediation model is discussed separately.

As predicted in our hypothesis 2, expatriates' international assignments experience moderates the association between stressors during COVID-19 and psychological resilience. Specifically, the negative predicting role of stressors during COVID-19 on psychological resilience is significant slowdown among those with richer international assignments experience; in contrast, for those with less international assignments experience, the relation between stressors during COVID-19 and psychological resilience is stronger. This is consistent with the point of view of resource conservation theory (67). Expatriate experience is an essential resource for individual international construction expatriates. When employees encounter stressors, the more experienced one can fully mobilize their resources (71). It is easier for them to find ways and attitudes to deal with the incident from their experience, thereby improving their ability to return to normal. Nevertheless, international construction is a highly uncertain environment (72); especially during the outbreak and duration of the COVID-19 pandemic, expatriates' feelings about home are amplified (73). This is also related to the feelings toward home in Chinese traditional culture (74). However, the continuation of the epidemic has made the road home extremely difficult. At this time, international assignments experience can fully adjust the ability of expatriates to regulate stressors and quickly recover to a well-adjusted state. This suggests that experience is one of the buffering factors for individual psychological resilience of expatriates to regulate stress.

As predicted in our hypothesis 3, expatriates' international assignments experience moderates the second mediation path from psychological resilience to PHQ, GAD, and PSS. The moderating role of expatriates' international assignments experience can be explained by the combined action of expatriates' international assignments experience and psychological resilience on PHQ, GAD, and PSS (60). Rossi et al. (12) find that age plays a moderating role between resilience, depression, and anxiety. Generally speaking, older people have more work experience. Expatriates with high psychological resilience are less likely to have depression and other emotions (75) because they have a more vital ability to adjust themselves to external events. This phenomenon is evident among experienced expatriates. These people can find similar experiences from their existing experiences and draw inferences from one instance (76). They know better how to improve their ability to adapt to crises to avoid unhealthy psychological states such as depression and anxiety. At the same time, the existing experience allows them to know the possible results after the occurrence of stressors to reduce their fear of the unknown future, which is very important for emotional stability and mental health.

When comparing the moderating effect in the two stages, it is more significant between stressors and psychological resilience. This result indicate that psychological resilience can be affect significantly by individual resources. Expatriates can take more measures to deal with adversities and adapt to health status. However, once the level of psychological resilience decreases and causes mental health problems, the moderating effect of experience is less significant.

### Practical implication

Our research has practical value for international construction enterprises and managers of international construction projects, including three aspects. First, managers accurately understand the psychological health of expatriates under the normalization of COVID-19 and formulate intervention and help plans in time. Secondly, managers are more transparent that priority should be given to those with rich expatriate experience when selecting expatriates. In emergencies, experienced people can do an excellent job in self-psychological adjustment. Finally, managers should pay more attention to the psychological resilience of expatriates, which negatively affects the psychologically unhealthy states of expatriates, such as depression, anxiety, and stress. The individual's psychological resilience can be improved. Managers can prioritize those with high psychological resilience to work abroad and formulate improvement plans to help expatriates.

### Limitations and future research

There are several limitations to this study. First, due to the limitations of cross-sectional data, the results of this study have limitations. The follow-up research can increase the data collection of profile and psychological experiment. Second, this study makes a subjective evaluation of self-perception. Although this method is widely used in a large number of studies, there may be a deviation between participants' self-perception and the actual state. In the future, physical measurement tools can be used to assist in the evaluation of psychological and emotional states. Finally, all the participants are Chinese. The cultural background of different countries may lead to differences in their psychological resilience. At the same time, the social environment of the host country may also have effect on physical and mental health of expatriates. Therefore, our findings may not be applicable to expatriates from other cultural backgrounds. In future research, we will cooperate with more international contractors to collect data to verify the research results and take specific measures to improve the psychological resilience of expatriates.

## Conclusion

Based on our analysis and discussion, the current study suggests that expatriates' psychological resilience mediates the relationship between stressors during COVID-19 and mental health. International assignments experience moderates the first and second half of the mediation process. Furthermore, stressors affect mental health through expatriates' psychological resilience.

The research provided a theoretical and empirical basis for understanding the relationship between stressors during COVID-19 and expatriates' mental health. According to the moderated mediating model, stressors negatively predicted psychological resilience, and psychological resilience negatively predicted depression, anxiety, and perceived stress. Moreover, when expatriates have high international assignments experience, their psychological resilience level had a significant descending trend as the increased COVID-19 related stressors. On the other hand, when expatriates have low international assignments experience, the effect of psychological resilience on expatriates' depression, anxiety, and perceived stress level is significantly weakened with the increased level of psychological resilience. The results also indicate that the mental health of expatriates in international projects not only depends on individual resources, but also requires resources at the project team and organization level. In addition, we can effectively intervene in the impact of stressors on mental health by improving expatriates' psychological resilience during COVID-19. Meanwhile, managers can prioritize international expatriates with rich experience working on overseas projects in the post epidemic era.

## Data availability statement

The raw data supporting the conclusions of this article will be made available by the authors, without undue reservation.

## Author contributions

LG: writing—original draft, writing—review and editing, formal analysis, and validation. XD: conceptualization, supervision, funding acquisition, and resources. WY: software and visualization. JF: investigation and data collection. All authors contributed to the article and approved the submitted version.

## Funding

This work was supported by the National Natural Science Foundation of China (Grant Nos. 72171048 and 72101053), the Scientific Research Foundation of Graduate School of Southeast University (Grant No. YBPY2130), and Postgraduate Research & Practice Innovation Program of Jiangsu Province (Grant No. KYCX21_0167).

## Conflict of interest

JF was employed by SINOPEC Engineering (Group) Co. Ltd. (SEG). The remaining authors declare that the research was conducted in the absence of any commercial or financial relationships that could be construed as a potential conflict of interest.

## Publisher's note

All claims expressed in this article are solely those of the authors and do not necessarily represent those of their affiliated organizations, or those of the publisher, the editors and the reviewers. Any product that may be evaluated in this article, or claim that may be made by its manufacturer, is not guaranteed or endorsed by the publisher.

## Appendix

**Appendix A:**

**Code**	**Construct and items**	**Sources**
Psychological resilience (PR)		(56 )
PR01	Able to adapt to change.	
PR02	Can deal with whatever comes.	
PR03	See the humorous side of things.	
PR04	Coping with stress strengthens.	
PR05	Tend to bounce back after illness or hardship.	
PR06	Can achieve the goals.	
PR07	Under pressure, focus and think clearly.	
PR08	Not easily discouraged by failure.	
PR09	Think of self as strong person.	
PR10	Can handle unpleasant feelings.	
Patient health questionnaire (PHQ-9)		(57)
PHQ 01	Little interest or pleasure in doing things	
PHQ 02	Feeling down, depressed, or hopeless	
PHQ 03	Trouble falling or staying asleep, or sleeping too much	
PHQ 04	Feeling tired or having little energy	
PHQ 05	Poor appetite or overeating	
PHQ 06	Feeling bad about yourself – or that you are a failure or have let yourself or your family down	
PHQ 07	Trouble concentrating on things, such as reading the newspaper or watching television	
PHQ 08	Moving or speaking so slowly that other people could have noticed?	
PHQ 09	Thoughts that you would be better off dead or of hurting yourself in some way	
Generalized anxiety disorder questionnaire (GAD-7)		(58)
GAD01	Feeling nervous, anxious or on edge	
GAD02	Not being able to stop or control worrying	
GAD03	Worrying too much about different things	
GAD04	Trouble relaxing	
GAD05	Being so restless that it is hard to sit still	
GAD06	Becoming easily annoyed or irritable	
GAD07	Feeling afraid as if something awful might happen	
Perceived stress scale (PSS-10)		(59)
PSS01	In the last month, how often have you been upset because of something that happened unexpectedly?	
PSS02	In the last month, how often have you felt that you were unable to control the important things in your life?	
PSS03	In the last month, how often have you felt nervous and “stressed”?	
PSS04	In the last month, how often have you dealt successfully with irritating	
PSS05	In the last month, how often have you felt that you were effectively coping with important changes were occurring in your life?	
PSS06	In the last month, how often have you felt confident about your ability to handle your personal problems?	
PSS07	In the last month, how often have you felt that things were going your way?	
PSS08	In the last month, how often have you found that you could not cope with all the things that you had to do?	
PSS09	In the last month, how often have you been able to control irritations in your life?	
PSS10	In the last month, how often have you felt that you were on top of things?	
